# Preoperative Intravitreal Conbercept Injection Reduced Both Angiogenic and Inflammatory Cytokines in Patients With Proliferative Diabetic Retinopathy

**DOI:** 10.1155/2024/2550367

**Published:** 2024-09-14

**Authors:** Zijing Huang, Li Jia Chen, Dingguo Huang, Jingsheng Yi, Zhiying Chen, Peimin Lin, Yifan Wang, Jianlong Zheng, Weiqi Chen

**Affiliations:** ^1^ Joint Shantou International Eye Center of Shantou University and The Chinese University of Hong Kong, Shantou, Guangdong, China; ^2^ Department of Ophthalmology & Visual Sciences The Chinese University of Hong Kong, Sha Tin, New Territories, Hong Kong; ^3^ Fifth Clinical Institute Shantou University Medical College, Shantou, Guangdong, China

**Keywords:** conbercept, cytokines, diabetic retinopathy, inflammation, PlGF

## Abstract

**Aims:** To investigate the impact of intravitreal injection of conbercept, a recombinant fusion protein with decoy receptors for the vascular endothelial growth factor (VEGF) family, on intraocular concentrations of angiogenic and inflammatory mediators in patients with proliferative diabetic retinopathy (PDR), analyzed its potential impact on surgical outcomes.

**Methods:** Forty eyes from 40 patients with PDR were included in this prospective study. Patients received intravitreal injection of conbercept followed by vitrectomy or phacovitrectomy in 1 week. Aqueous humor samples were collected before and 1 week after the conbercept injection. The concentrations of angiogenic and inflammatory cytokines and chemokines were measured by flow cytometry. Follow-up clinical data were collected and analyzed.

**Results:** Intravitreal conbercept injection significantly decreased aqueous concentrations of VEGF (325.5 (baseline) versus 22.3 pg/mL (postinjection), *p* < 0.0001), PlGF (39.5 versus 24.5 pg/mL, *p* < 0.0001), and PDGF-A (54.1 versus 47.0 pg/mL, *p* = 0.0016), while no impact on bFGF levels. For inflammatory mediators, the concentration of TNF-*α* (0.79 versus 0.45 pg/mL, *p* = 0.0004) and IL-8 (180.6 versus 86 pg/mL, *p* < 0.0001) were decreased, while IL-6 (184.1 versus 333.7 pg/mL, *p* = 0.0003) and IL-10 (1.1 versus 1.5 pg/mL, *p* = 0.0032) were increased. No significant changes in IFN-*γ* or MCP-1 were detected. Three months after surgery, the mean best-corrected visual acuity improved from a baseline of 1.8 ± 0.1 logMAR to 0.7 ± 0.1 logMAR (*p* < 0.0001), with 36 eyes (90%) achieving an improvement of visual function.

**Conclusions:** Intravitreal conbercept injection presents dual effects of antiangiogenesis and anti-inflammation and can be served as an adjuvant treatment to vitrectomy for PDR patients.

## 1. Introduction

Proliferative diabetic retinopathy (PDR) is a leading cause of severe visual loss in diabetes population [1]. In China, a significant number of individuals with diabetes experience poor blood sugar control and delayed ocular intervention. Regrettably, they tend to seek medical intervention only after the onset of severe complications, such as vitreous hemorrhage and tractional retinal detachment [2]. Ample evidence has demonstrated that vascular endothelial growth factor (VEGF or VEGF-A) plays a pivotal role in the development of retinal neovascular diseases. Anti-VEGF agents, such as ranibizumab, have emerged as novel and effective approaches to the treatment of these devastating diabetic complications [3, 4]. Studies using anti-VEGF agents alone or as an adjunct to other therapies have shown better outcomes and/or reduced side effects in the management of aggressive PDR [5, 6].

Studies have indicated, however, that anti-VEGF treatment may lead to compensatory VEGF-independent angiogenesis by expression of genes encoding other VEGF-related and nonrelated angiogenic factors, such as placental growth factor (PlGF), basic fibroblast growth factor (bFGF), and platelet-derived growth factor-A (PDGF-A), which partially explain the less-than-optimal response observed in certain patients undergoing anti-VEGF treatment [7, 8]. In addition to microvascular abnormalities, growing clinical evidence has demonstrated inflammation as another key player and therapeutic target in diabetic retinopathy pathology. Cellular events, such as leukostasis, gliosis, and increased expression of inflammatory mediators, are involved in the pathogenesis of PDR [9, 10]. These inflammatory markers include tumor necrosis factor *α* (TNF-*α*); interleukin (IL)-6, IL-8, IL-10; interferon-*γ* (IFN-*γ*); intercellular adhesion molecule-1 (ICAM-1); and monocyte chemoattractant protein-1 (MCP-1) [11, 12].

Conbercept is a recombinant fusion protein with a high affinity to all isoforms of VEGF and PlGF [13]. Studies have indicated preoperative intravitreal injection of conbercept as a promising strategy for the surgical treatment of complicated PDR, facilitating easier and safer surgery regarding less risk of intraoperative and early postoperative hemorrhage, reducing the need for silicone oil and relaxing retinotomy, and leading to better anatomical and functional outcomes [14, 15]. The effects of intravitreal conbercept injection on other angiogenic factors during the treatment of PDR remained not fully understood. Furthermore, conbercept has shown potential anti-inflammatory effects through binding to PlGF, which is a VEGF receptor-1 ligand and plays a role in the modulation of inflammatory response [16]. Thus, it would be interesting and important to investigate the effect of conbercept on proinflammatory cytokines during the treatment of PDR.

In this study, we investigated the effect of preoperative intravitreal conbercept injection on angiogenic and inflammatory cytokines and analyzed its potential impact on surgical outcomes.

## 2. Materials and Methods

### 2.1. Subjects and Ethics Approval

This prospective and interventional study conformed to the tenets of the Declaration of Helsinki. The study was conducted in compliance with a suitable accredited institutional review board from the Ethics Committee of Joint Shantou International Eye Center (JSIEC) of Shantou University and the Chinese University of Hong Kong (No. EC 20221115(10)-P11). Informed consent was obtained from every patient by signing a consent form after an explanation of the nature and possible consequences of the study. Consecutive patients from March to November 2022 who met the following criteria were included: (1) patients diagnosed with active PDR in at least one eye, characterized by nonclearing vitreous hemorrhage and/or tractional retinal detachment, and required surgery; (2) only one eye of each patient was included in the study and the included eyes had no history of vitreoretinal surgery and had not been treated with cataract extraction or intravitreal injection of anti-VEGF agents/corticosteroids within 3 months. Exclusion criteria included (1) combined with other ocular disorders, such as retinal vein occlusion, macular hole, age-related macular degeneration, and glaucoma; and/or (2) confounding systemic diseases, such as uncontrolled hypertension and heart failure.

### 2.2. Data Collection

Patients underwent a detailed medical history taking and comprehensive ocular examination before intravitreal injection of conbercept, including best corrected visual acuity (BCVA) using the International Standard Visual Acuity Chart, intraocular pressure (IOP) using noncontact tonometer, slit lamp examination, and dilated fundus examination. B-scan was performed to assess the degree of vitreous hemorrhage and determine whether vitreous proliferation and/or retinal detachment was present in patients with poor fundal view. Laboratory-based testing, including fasting blood glucose levels, hemoglobin A1c (HbA1c), hemoglobin, blood lipid, and renal function tests, were collected and analyzed 1 day before conbercept injection. Follow-up data including postoperative BCVA, IOP, optical coherence tomography (OCT)-based macular thickness, and complications 1 week after intravitreal conbercept injection, 1 month after surgery, and 3 months after surgery, were collected and analyzed.

### 2.3. Aqueous Sample Collection and Intravitreal Injection

Undiluted aqueous samples were harvested twice in each patient: (1) before intravitreal injection of conbercept and (2) before the phacovitrectomy procedure 1 week after intravitreal injection of conbercept. A 30-gauge fine needle was gently inserted into the temporal anterior chamber and approximately 100–200 *μ*L of aqueous humor was aspirated. The samples were immediately transferred to a sterile plastic tube and stored at −80°C until further analysis. All patients then received an intravitreal injection of conbercept (KH902; Chengdu Kanghong Biotech Co. Ltd., China) as an off-label treatment for PDR. After topical anesthesia, conbercept (0.5 mg/0.05 mL) was administered intravitreally using a 30-gauge needle 3.5 mm posterior to the limbus at the inferotemporal site.

### 2.4. Surgical Procedures

Standard 23-gauge three-port pars plana vitrectomy (PPV) was performed 1 week after the intravitreal injection for all the participants. Endolaser panretinal photocoagulation (PRP) was performed, followed by air (*n* = 32) or silicone oil tamponade (*n* = 8) at the surgeon's discretion. Intraocular diathermy was used in 14 eyes (35%) to treat active intraocular bleeding. Triamcinolone acetonide (2 mg in 0.05 ml) was injected into the vitreous cavity at the end of the surgery. For eyes with significant cataract, phacoemulsification (*n* = 29) with implantation of intraocular lens (*n* = 25) was performed combined with vitrectomy.

### 2.5. Measurement of Proangiogenic and Inflammatory Cytokines

The measurement of cytokines was conducted by a qualified third-party testing institution (Beijing Giantmed Medical Diagnostics Lab). All samples were coded, and the testing was performed in a blind manner. The concentrations of 10 angiogenic and inflammatory cytokines/chemokines were measured by cytometric bead array as previously described [17]. Briefly, 25 *μ*L of the centrifuged sample supernatant was added to the prepared beads working solution, which contained 22 *μ*L of beads diluent and 0.5 *μ*L of beads. The sample was thoroughly mixed and incubated in the dark for 1 h. Subsequently, 25 *μ*L of the PE working solution (22 *μ*L reagent diluent and 0.5 *μ*L of PE) was added to the sample. This mixture was incubated in the dark for an additional 2 h to form the “beads-antibody-sample antigen-PE antibody” complex. After washing with the wash buffer, the samples were analyzed using a flow cytometer (FACS CantoTMII, BD Bioscience, San Jose, CA, United States). The software FCAP Array v3 (BD Bioscience, San Jose, CA, United States) was used to generate a standard curve and calculate the concentration values. The cytokines/chemokines include VEGF-A, PlGF, PDGF-A, bFGF, MCP-1, IFN-*γ*, TNF-*α*, IL-6, IL-8, and IL-10. Concentrations lower than the lower limit of detection were defined as nonmeasurable and were excluded from further analysis.

### 2.6. Statistical Analysis

All statistical analyses were carried out using SPSS V.18.0 (SPSS, Inc, Chicago, Illinois, United States). The Wilcoxon test was used to compare the aqueous concentration of angiogenic and inflammatory cytokines before and after conbercept injection. The Pearson parametric and the Spearman nonparametric correlation analyses were used to analyze the correlation between the changes in levels of VEGF/PlGF and other mediators before and after conbercept injection. The Wilcoxon rank-sum test was used for comparison of BCVA and IOP, respectively, at baseline and several time points after surgery. BCVA was converted to logMAR before statistical analysis. Data were presented as mean ± standard error of mean (SEM). Statistical significance was accepted at *p* < 0.05.

## 3. Results

### 3.1. Demographics

A total of 40 eyes from 40 patients with PDR were included in this work, with a mean age of 52.4 ± 11.0 years (range 24–69 years). There were 16 males (40%) and 24 females (60%). Among the 40 eyes, there were 27 right eyes (67.5%) and 13 left eyes (32.5%). The mean duration of diabetes at study recruitment was 8.7 ± 5.3 years. Twenty-nine patients (72.5%) had varying degrees of systemic hypertension. One day before intravitreal conbercept injection, the mean fasting blood sugar was 9.9 ± 2.8 mmol/L, and the percentage of HbAlc was 8.7 ± 1.7%. The average hemoglobin level at presentation was 121.2 ± 17.8 g/L, and 5 patients (12.5%) were diagnosed with anemia (hemoglobin level < 120 g/L). The level of blood urea nitrogen was 9.3 ± 5.1 mmol/L, and the serum creatinine was 107.1 ± 70.8 *μ*mol/L, and 9 patients (22.5%) had previously been diagnosed with renal dysfunction. The mean triglyceride level was 2.3 ± 1.5 mmol/L and cholesterol level 5.5 ± 1.5 mmol/L. Twenty-nine patients (72.5%) had a history of hypertension while 8 patients (20%) had a history of hyperlipidemia.

Upon ocular examination, 5 eyes (12.5%) had previous laser treatment. Among them, 3 eyes underwent PRP, while the other 2 eyes underwent partial retinal laser photocoagulation due to vitreous hemorrhage. One eye (2.5%) received an intravitreal conbercept injection 6 months before study enrolment, 36 eyes (90%) had various degrees of vitreous hemorrhage, and 6 eyes (15%) had a tractional retinal detachment. No included eyes had a history of neovascular glaucoma. Thirty-six eyes (90%) were diagnosed with varying degrees of cataract, among which 29 eyes (72.5%) had combined phacoemulsification with vitrectomy due to significant lens opacification, 1 eye had normal lens, and the other 3 eyes were pseudophakic at baseline.  Table 1 summarizes the demographics of patients.

### 3.2. Angiogenic Factors

**Figure 1:**
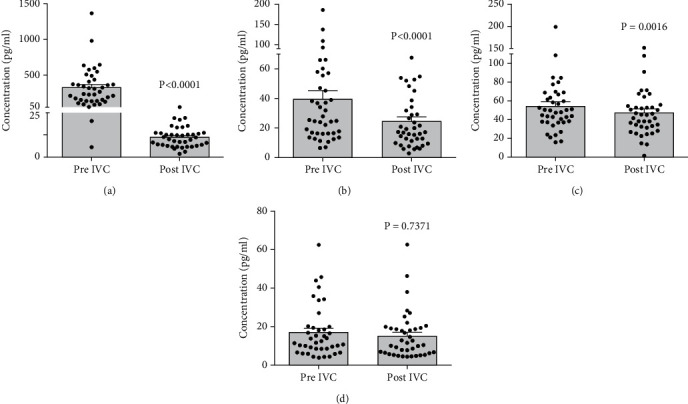
The changes in aqueous concentrations of angiogenic cytokines before and 1 week after intravitreal conbercept injection in patients with proliferative diabetic retinopathy. Note that conbercept treatment significantly reduced the levels of (a) VEGF, (b) PlGF, and (c) PDGF-A while having no impact on (d) bFGF secretion. IVC, intravitreal injection of conbercept. Data were presented as scatter plot and median ± standard error of mean.

Before conbercept injection, the median concentrations of angiogenic factors in aqueous humor were as follows: VEGF-A 325.5 ± 42.1 pg/mL, PlGF 39.5 ± 5.9 pg/mL, PDGF-A 54.1 ± 4.9 pg/mL, and bFGF 17.0 ± 2.1 pg/mL. One week after conbercept injection, the aqueous concentration of VEGF-A, PlGF, PDGF-A, and bFGF was 22.3 ± 1.8 pg/mL, 24.5 ± 2.9 pg/mL, 47.0 ± 4.2 pg/mL, and 15.2 ± 1.9 pg/mL, respectively. Intravitreal conbercept injection markedly decreased the levels of VEGF-A (*p* < 0.0001), PlGF (*p* < 0.0001), and PDGF-A (*p* = 0.0016), while no significant impact on the level of bFGF (*p* = 0.7371) in the aqueous humor of PDR patients (Figure 1).

### 3.3. Inflammatory Cytokines

**Figure 2:**
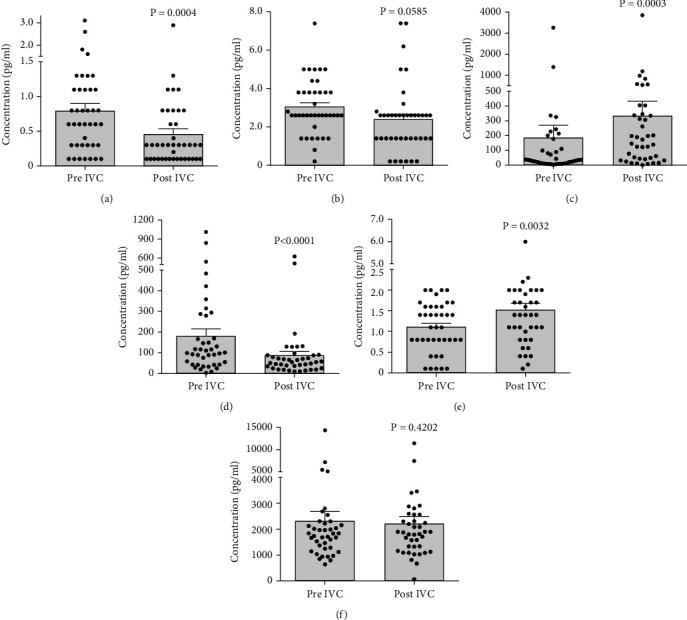
The changes in aqueous concentrations of proinflammatory cytokines/chemokines before and 1 week after intravitreal conbercept injection in patients with proliferative diabetic retinopathy. Conbercept treatment significantly reduced the levels of (a) TNF-*α* and (d) IL-8, increased the levels of (c) IL-6 and (e) IL-10, and had no impact on (b) IFN-*γ* and (f) MCP-1 expression. IVC, intravitreal injection of conbercept. Data were presented as scatter plot and median ± standard error of mean.

Before conbercept injection, the median concentrations of inflammatory cytokines and chemokines in aqueous humor were as follows: TNF-*α* 0.79 ± 0.1 pg/mL, IFN-*γ*3.0 ± 0.2 pg/mL, IL-6 184.1 ± 86.8 pg/mL, IL-8 180.6 ± 34.5 pg/mL, IL-10 1.1 ± 0.1 pg/mL, and MCP-1 2303 ± 369 pg/mL. One week after conbercept injection, the aqueous concentrations of these cytokines and chemokines were TNF-*α*0.45 ± 0.1 pg/mL, IFN-*γ*2.4 ± 0.3 pg/mL, IL-6 333.7 ± 99.9 pg/mL, IL-8 86.0 ± 19.1 pg/mL, IL-10 1.5 ± 0.2 pg/mL, and MCP-1 2198 ± 300 pg/mL. The levels of TNF-*α* and IL-8 were significantly decreased (*p* = 0.0004 and *p* < 0.0001, respectively), while IL-6 and IL-10 were increased (*p* = 0.0003 and *p* = 0.0032, respectively). No significant differences in the levels of IFN-*γ* or MCP-1 were found before and after conbercept administration (*p* = 0.0585 and *p* = 0.4202, respectively) (Figure 2).

### 3.4. Correlation Between Angiogenic and Proinflammatory Cytokines

There was a positive correlation between the changes in levels of VEGF-A and PlGF (*p* < 0.001, correlation coefficient 0.649, Spearman correlation), VEGF-A and PDGF-A (*p* = 0.010, correlation coefficient 0.404, Pearson correlation), VEGF-A and TNF-*α* (*p* < 0.001, correlation coefficient 0.682, Spearman correlation), PlGF and PDGF-A (*p* = 0.002, correlation coefficient 0.437, Pearson correlation), and PlGF and TNF-*α* (*p* = 0.005, correlation coefficient 0.482, Spearman correlation), while IL-6 level was found negatively correlated with VEGF-A (*p* = 0.004, Correlation coefficient −0.442, Spearman correlation). No correlation was found between the changes in other cytokines during conbercept treatment.

### 3.5. Follow-Up Clinical Findings

**Figure 3:**
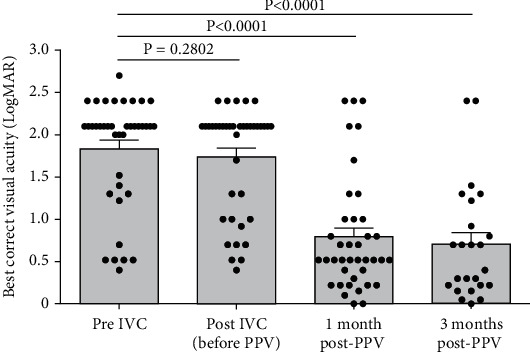
Best corrected visual acuity during the treatment process. A significant improvement in BCVA was observed after surgery as compared to baseline. IVC, intravitreal injection of conbercept; PPV, pars plana vitrectomy. Data were presented as scatter plot and median ± standard error.

The mean BCVA was 1.83 ± 0.10 logMAR before intravitreal conbercept injection and was 1.74 ± 0.10 at 1 week after conbercept injection (*p* = 0.2802). One month after the phacovitrectomy procedures, the BCVA was 0.79 ± 0.11 logMAR (*p* < 0.0001, compared to baseline). Three months after the surgery, the BCVA was 0.70 ± 0.14 logMAR (*p* < 0.0001, compared to baseline) (Figure 3).

The IOP at baseline was measured as 14.7 ± 2.9 mmHg and was documented at 14.5 ± 3.5, 14.5 ± 3.6, and 15.6 ± 6.2 mmHg at 1 week after preoperative conbercept injection, at 1 month after surgery, and at 3 months after surgery, respectively. No statistically significant variations were observed among these recorded values. None of the eyes developed neovascular glaucoma during the follow-up period.

Due to the presence of severe vitreous hemorrhage, the feasibility of OCT measurements was limited to 15 eyes at baseline. In this subgroup, the central macular thickness showed a mean of 289.5 ± 211.1 *μ*m before treatment, 326.6 ± 201.5 *μ*m at 1 month after surgery, and 279.7 ± 136.1 *μ*m at 3 months after surgery, with no statistically significant changes observed. At 3 months after surgery, the average blood glucose level was measured at 9.0 ± 2.9 mmol/L, and the HbA1c stood at 8.1 ± 1.1%. These data exhibited no significant difference as compared to the baseline.

Within the 3-month postoperative timeframe, one eye (2.5%) developed a recurrent and nonclearing vitreous hemorrhage requiring a vitreous cavity lavage procedure. This intervention resulted in an improvement of BCVA, presenting from 1.85logMAR (finger counting) to 0.70logMAR (40/200 Snellen). In addition, two cases (5%) with preoperative tractional retinal detachment displayed recurrent retinal detachment, of which one received a subsequent surgical intervention to achieve retinal reattachment. The BCVA improved from 2.30logMAR (hand motion) to 1.00logMAR (20/200 Snellen) in the last follow-up. The other patient opted against further therapeutic procedures.

## 4. Discussion

In recent years, anti-VEGF drugs have been utilized as an adjuvant therapy to vitrectomy for PDR patients, with a view to mitigate intraoperative bleeding and early postoperative rebleeding and to facilitate shorter operation time [14, 18]. However, in vitro studies have revealed that anti-VEGF administration resulted in alterations of other VEGF-dependent and -independent proangiogenic factors, such as PlGF, PDGF-A, bFGF, and proinflammatory factors, such as interleukins and TNF-*α* [7, 8]. The compensatory increase in these factors may conceal the therapeutic effects of anti-VEGF therapy. Hence, investigating the changes in these vascular and inflammatory mediators following anti-VEGF therapy is important for further understanding of the molecular mechanism and developing treatment strategies.

In this study, intravitreal administration of conbercept significantly reduced the concentrations of TNF-*α* and IL-8 in the aqueous humor of PDR patients. TNF-*α* is a crucial inflammatory mediator that increases vascular endothelial cell permeability and promotes the synthesis and release of other proinflammatory cytokines [19], while IL-8 promotes the inflammatory response through chemotaxis and activation of neutrophils and T lymphocytes [20]. Importantly, IL-8 also exerts a proangiogenic effect in hypoxia-induced retinal neovascularization [21]. Conbercept injection also increased the expression of IL-10, a recognized anti-inflammatory and immunosuppressive factor that regulates cell growth and differentiation [22]. However, IL-6, another core inflammatory cytokine, was found significantly upregulated after conbercept injection. This is consistent with a previous study in which anti-VEGF injection in PDR patients led to a transient decrease in IL-6 concentrations in the vitreous on the 3^rd^ day, followed by a significant increase on the 7^th^ day, even exceeding the preinjection level [23]. Compensatory elevation of IL-6 caused by anti-PlGF treatments may be a possible explanation. In addition, conbercept had no limited influence on the aqueous levels of IFN-*γ* and MCP-1, which is consistent with a previous study investigating the impact of conbercept on vitreous inflammatory cytokines in patients with diabetic macular edema [24]. Collectively, our data indicated the anti-inflammatory property of conbercept in PDR, although the effect may be partial and temporary. A combination of conbercept and anti-inflammatory agents, such as dexamethasone, can be considered when necessary to achieve better anti-inflammatory actions. It is also recommended to administer a small dose of triamcinolone acetonide at the end of the surgery in order to mitigate postoperative inflammation.

The anti-inflammatory property of conbercept has been demonstrated in inflammatory disease models [25], which is thought to be achieved through targeting PlGF [26]. Specifically, PlGF binds to VEGF receptor-1 (VEGFR-1), leading to activation of the PI3 kinase/AKT and ERK-1/2 signaling pathways and subsequent recruitment and chemotaxis of mononuclear macrophages, as well as polarization of macrophages towards an M1 proinflammatory phenotype [26]. The activated macrophages promote upregulation of VEGFR-1 and PlGF to form an autocrine positive feedback loop, which further exacerbates the inflammatory cascade [27]. In this study, we found a positive correlation between the changes in PlGF and TNF-*α* concentrations subsequent to conbercept treatment, suggesting a potential association between PlGF and inflammation.

Studies have demonstrated that intravitreal administration of conbercept and VEGFR-1 inhibitors can alleviate retinal inflammation in the mouse models of choroidal neovascularization and diabetes [28, 29]. On the other hand, anti-VEGF or anti-VEGFR-2 therapies failed to produce comparable effects [28, 29]. One possible explanation is that anti-VEGF monotherapy results in compensatory upregulation of PlGF [30], which activates the PlGF/VEGFR-1-dependent inflammatory pathway. These findings, together with ours, suggested that combined PlGF and VEGF inhibition may have a preferable benefit than simply targeting VEGF, particularly in disorders like diabetic retinopathy where inflammation becomes increasingly activated during the disease process.

In this study, we also investigated the impact of conbercept on the concentrations of other proangiogenic factors, PDGF-A and bFGF, known to play crucial roles in promoting angiogenesis and fibrosis processes [31, 32]. The results showed that the PDGF-A level was significantly downregulated, whereas bFGF showed a slight decrease that did not reach statistical significance. The exact mechanism by which conbercept affects these cytokines remains unclear. Previous studies have demonstrated that VEGF competitively inhibits the binding and activation of PDGF-A to PDGF receptor (PDGFR) [33, 34]. Therefore, VEGF inhibition may conversely promote the binding, internalization, and degradation of PDGF-A and PDGFR, leading to a reduction in the free PDGF-A molecule. Anti-VEGF therapy can also alleviate proliferative vitreoretinopathy by inhibiting the PDGFR/PI3K/Akt signaling pathway [35]. In addition, the change in PDGF-A level was positively correlated with both VEGF and PlGF after conbercept injection, indicating the involvement of VEGF/PlGF in the regulation of PDGF signaling. In addition, researches have revealed a crosstalk between the bFGF and VEGF signaling pathways. For instance, VEGF and bFGF can regulate the expression levels of each other's receptors, and both promote angiogenesis through the downstream MAPK/ERK signaling pathway [36, 37]. In addition, anti-VEGF therapy may induce fibrous vascular membrane contraction, with bFGF potentially playing a role in this process [38]. However, in this study, conbercept injection had no significant effect on intraocular bFGF levels. One possible reason for this could be the short detection interval, which may not have allowed sufficient time for the effect to manifest. Collectively, conbercept attenuated the upregulation of several proangiogenic factors, suggesting a wider action spectrum of this fusion protein.

Interestingly, factors such as gender, age, and insulin use have been reported to affect cytokine levels within the body, particularly proinflammatory factors [39–42]. In this study, we compared the differences in changes of intraocular cytokine levels before and after conbercept treatment between males and females, patients aged < 45 years and > 45 years, and patients with and without insulin treatment. Unfortunately, there were no significant differences between these subgroups (Figure S1). Nevertheless, females appear to present smaller decreases in several cytokines including VEGF, PlGF, PDGF-A, bFGF, and TNF-*α* after treatment, suggesting that conbercept may have a pronounced effect on improving intraocular microenvironment in males. Further investigation with a larger sample size is warranted.

In this study, the mean BCVA significantly improved from a baseline of 1.83 ± 0.10 logMAR to a 3-month postoperation of 0.70 ± 0.14 logMAR, with 36 eyes (90%) achieving an improvement of visual function. Silicone oil was required for 8 of the 40 eyes (20%), while diathermy was needed for 14 eyes (35%). Postoperatively, one eye (2.5%) experienced nonclearing vitreous hemorrhage, and 2 eyes (5%) developed recurrent retinal detachment. These findings were comparable to previous studies [6], suggesting favorable outcomes within our cohort. Importantly, we found that adjunctive conbercept treatment not only reduced the levels of intraocular VEGF and PlGF but also exerted a modulating influence on the concentrations of several proinflammatory factors, although the reasons for this remain not fully understood. Our results provide a theoretical basis for the effectiveness of conbercept as preoperative adjuvant therapy for PDR and also highlight the importance of anti-inflammation, apart from anti-VEGF, in the management of PDR.

The findings of this study hold potential clinical applicability. Firstly, conbercept as an adjunctive therapy could orchestrate multiple intraocular angiogenic and inflammatory factors in the short term, creating a favorable microenvironment for the subsequent vitrectomy procedures. Secondly, the upregulation of certain proinflammatory factors (such as IL-6) following conbercept treatment suggests using conbercept alone may not provide long-term and robust anti-inflammatory effects. A combination therapy of anti-VEGF with other anti-inflammatory agents, such as corticosteroids, tyrosine kinase inhibitors, or anti-TNF-*α* monoclonal antibodies, could be a potential direction for future research. Finally, further research is needed to clarify the mid to long-term effects of conbercept, as well as other anti-VEGF drugs such as ranibizumab and aflibercept, on intraocular cytokines, thereby exploring optimal intervention strategies.

This study was limited by its relatively small sample size, where we chose only one observation time point and failed to understand the dynamic changes of cytokines after anti-VEGF treatment. In addition, we collected aqueous humor for analysis due to the inability to obtain vitreous samples during the intravitreal conbercept treatment procedure. Given that the vitreous is anatomically closer to the retina, vitreous fluid may better reflect the actual levels of cytokines in PDR patients.

**Figure 4:**
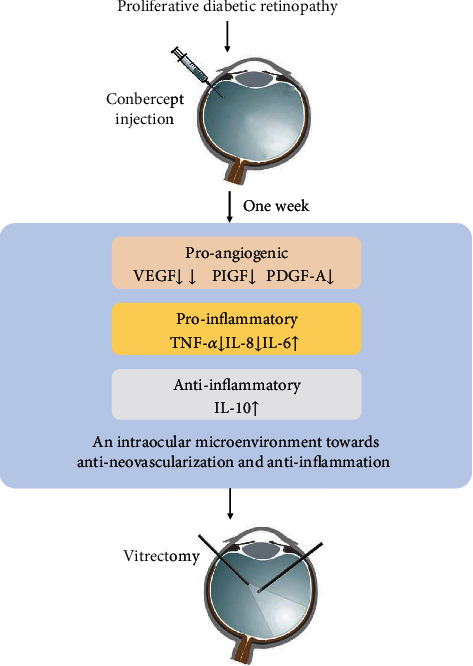
A summary model of this study. A preoperative intravitreal injection of conbercept administered 1 week before vitrectomy in patients with proliferative diabetic retinopathy effectively decreased the concentration of multiple proangiogenic factors and modulated the inflammatory microenvironment within the eye. However, the expression of IL-6, one of the primary inflammatory mediators, was increased after conbercept injection.

In summary, intravitreal conbercept injection in PDR patients reduced the levels of various proangiogenic factors, including VEGF-A, PlGF, and PDGF-A. Additionally, it significantly downregulated inflammatory cytokines TNF-*α* and IL-8 and upregulated anti-inflammatory IL-10. The anti-inflammatory effect of conbercept might be attributed to its antagonistic action on PlGF. Our results provide a rationale for considering conbercept as an adjuvant treatment for PDR patients (Figure 4). Moreover, anti-PlGF provides an attractive therapeutic target that exhibits dual effects of antiangiogenesis and anti-inflammation.

## Figures and Tables

**Table 1:** Demographics of patients.

**Items**	**N** **u** **m** **b** **e** **r** ± **SD (%)**
Male	16 (40%)
Age (years)	52.4 ± 11.0
Type of diabetes	
Type I	3 (7.5%)
Type II	37 (92.5%)
Duration of diabetes (years)	8.7 ± 5.3
Use of insulin	18 (45%)
Systemic hypertension	29 (72.5%)
Laboratory findings	
Fasting blood glucose (mmol/L)	9.9 ± 2.8
Hemoglobin A1c (%)	8.7 ± 1.7
Hemoglobin (g/L)	121.2 ± 17.8
Blood urea nitrogen (mmol/L)	9.3 ± 5.1
Serum creatinine (*μ*mol/L)	107.1 ± 70.8
Triglyceride (mmol/L)	2.3 ± 1.5
Cholesterol (mmol/L)	5.5 ± 1.5
Ocular findings	
History of panretinal photocoagulation	5 (12.5%)
History of intravitreal conbercept injection	1 (2.5%)
Vitreous hemorrhage	36 (90%)
Tractional retinal detachment	6 (15%)
Cataract	36 (90%)

Abbreviations: SD, standard deviation.

## Data Availability

The datasets used and/or analyzed during the current study are available from the corresponding author on reasonable request.

## Nomenclature

BCVA: best corrected visual acuity
bFGF: basic fibroblast growth factor
IFN-*γ*: interleukin-gamma
IL: interleukin
IOP: intraocular pressure
MCP-1: monocyte chemoattractant protein-1
OCT: optical coherence tomography
PDGF-A: platelet-derived growth factor-A
PDR: proliferative diabetic retinopathy
PlGF: placental growth factor
TNF-*α*: tumor necrosis factor *α*
VEGF: vascular endothelial growth factor

## Supporting Information

Supporting Information Additional supporting information can be found online in the Supporting Information section. Figure S1. The differences in changes of intraocular cytokine levels before and after conbercept treatment between (A) males and females, patients (B) aged < 45 years and > 45 years, and patients (C) with and without insulin treatment. Data were presented as scatter plots and median ± standard error. ^∗^*p* < 0.05. ns, no significance.
