# Supplementary figures and images for: Preoperative Intravitreal Conbercept Injection Reduced Both Angiogenic and Inflammatory Cytokines in Patients With Proliferative Diabetic Retinopathy
Source: J Diabetes Res. 2024 Sep 14;2024:2550367. doi: 10.1155/2024/2550367 (PMC11416173; doi:10.1155/2024/2550367)

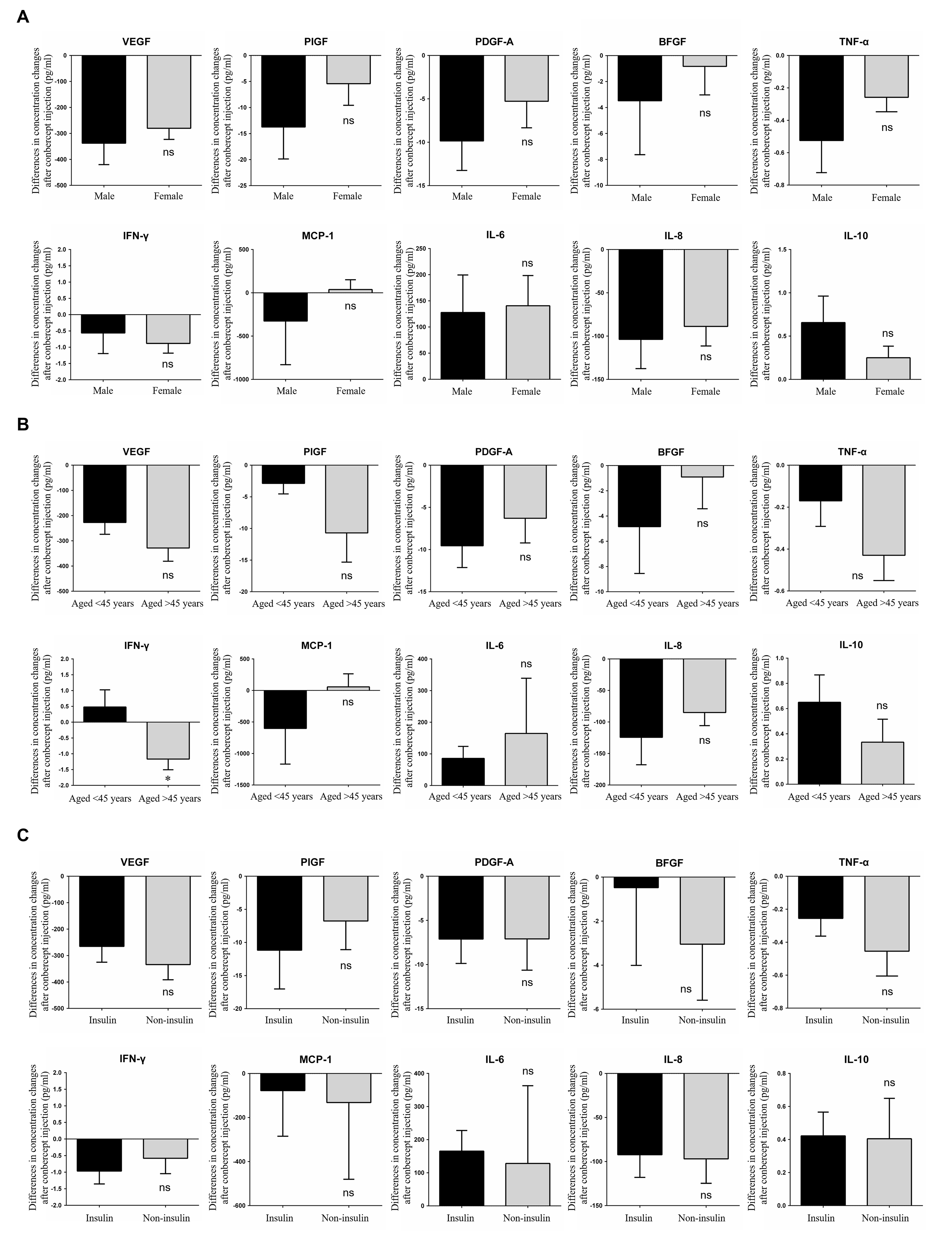

Supplement: Supporting Information — Additional supporting information can be found online in the Supporting Information section. Figure S1. The differences in changes of intraocular cytokine levels before and after conbercept treatment between (A) males and females, patients (B) aged < 45 years and > 45 years, and patients (C) with and without insulin treatment. Data were presented as scatter plots and median ± standard error. ∗p < 0.05. ns, no significance. [file 2550367.f1.jpg]
